# Improving behaviour in self-testing (IBIS): Study on frequency of use, consequences, information needs and use, and quality of currently available consumer information (protocol)

**DOI:** 10.1186/1471-2458-10-453

**Published:** 2010-08-03

**Authors:** Janaica EJ Grispen, Martine HP Ickenroth, Nanne K de Vries, Geert-Jan Dinant, Gaby Ronda, Trudy van der Weijden

**Affiliations:** 1 Department of General Practice, Faculty of Health, Medicine, and Life Sciences, Maastricht University, Maastricht, The Netherlands; 2 Department of Health Promotion, Faculty of Health, Medicine, and Life Sciences, Maastricht University, Maastricht, The Netherlands; 3 CAPHRI School for Public Health and Primary Care, Maastricht, The Netherlands

## Abstract

**Background:**

Self-tests are available to consumers for more than 25 conditions, ranging from infectious diseases to cardiovascular risk factors. Self-tests are defined as in-vitro tests on body materials such as blood, urine, faeces, or saliva that are initiated by consumers to diagnose a particular disorder or risk factor without involving a medical professional. In 2006, 16% of a sample of Dutch Internet users had ever used at least one self-test and 17% intended to use a self-test in the future. The objectives of this study are to determine (1) the frequency of self-test use, (2) the consumers' reasons for using or not using a self-test, (3) the information that is used by self-testers in the different self-test stages and the consumers' interpretation of the quality of this information, (4) the consumers' response to self-test results in terms of their confidence in the result, reassurance by the test result, and follow-up behaviour, (5) the information consumers report to need in the decision making process of using or not using a self-test, and in further management on the basis of the self-test result, and (6) the quality of the currently available consumer information on a selected set of self-tests.

**Methods:**

Mixed methods study with (1) a cross-sectional study consisting of a two-phase Internet-questionnaire, (2) semi-structured interviews with self-testers and consumers who intend to use a self-test, and (3) the assessment of the quality of consumer information of self-tests. The Health Belief Model and the Theory of Planned Behaviour will serve as the theoretical basis for the questionnaires and the interview topic guides.

**Conclusions:**

The self-testing area is still in a state of flux and therefore it is expected that self-test use will increase in the future. To the best of our knowledge, this is the first study which combines quantitative and qualitative research to identify consumers' information needs and use concerning self-testing, and the consumers' actual follow-up behaviour based on the self-test result, and simultaneously investigates the quality of the currently available consumer information. The results of this study will be used as an input in developing consumer information on self-testing.

## Background

A range of self-tests has become available to consumers in the Netherlands and elsewhere [1]. Self-tests are currently available for more than 25 conditions, ranging from infectious diseases to cardiovascular risk factors. Self-tests are not rarely used; 16% of a sample of Dutch Internet users indicated they had ever used at least one self-test in 2006, whereas 17% of those who had never used a self-test indicated they would probably or definitely use one in the future. The five most frequently used self-tests were tests for diabetes, cholesterol, allergies, urinary tract infection, and HIV [1].

We defined self-tests as in-vitro tests on body materials such as blood, urine, faeces, or saliva that are initiated by consumers to diagnose a particular disorder or risk factor. In other words, by using a self-test, consumers completely avoid the traditional health care systems. Pregnancy tests, home blood-pressure meters, and monitoring tests, e.g. serum glucose for patients with diabetes mellitus to follow-up on their disease and therapy, are examples of tests that are excluded by this definition. Self-tests can be subdivided into four distinct types that are directly accessible to consumers without the need for a preceding consultation with a doctor [1]. The first type of self-test concern self-tests for home use or the over-the-counter tests, in which the consumer is responsible for the execution and interpretation of the test as well as their follow-up behaviour. The second type of self-test comprises self-tests that are offered by an organization which carries out the test in public areas such as local supermarkets and the results are presented immediately. These tests are the so-called street-corner tests like the national cholesterol test offered by the Dutch Heart Association [2]. The third and fourth self-test categories include tests in which a consumer attends a laboratory facility to have body material taken (direct-access laboratory test) or sends body material to a laboratory where the test is executed (home-collect test), after which the result is sent to them by mail or via the Internet.

Four separate phases can be distinguished within the self-test procedure, namely (1) the decision process preceding the performance of a self-test, (2) the actual execution of the self-test (only applicable to over-the-counter tests), (3) the interpretation of the test-result, and (4) the consumers' follow-up behaviour based on the test-result.

In line with the current views on patient autonomy and self-management, self-diagnosis and self-testing can be used as a tool to take responsibility for one's own health [3,4] . However, the value of self-tests is heavily debated in the scientific literature. Proponents argue that self-testing increases testing rates, resulting in more timely diagnosis and treatment; self-testing is convenient and provides anonymity, and it promotes patient empowerment [5-8]. Opponents hold that at-risk populations do not use self-tests, self-tests entail relatively high costs, and testing without consulting a physician may result in adverse medical or psychological outcomes [5-7]. In view of this debate, effective consumer education seems essential in this new area, as self-tests are likely to become even more easily available and more widely used [1,3]. To the best of our knowledge, however, this is the first study to combine quantitative and qualitative research to identify consumers' information needs and use related to self-testing, and the consumers' actual follow-up behaviour based on the self-test result, and at the same time investigates the quality of the currently available consumer information. This study aims to identify potential problems and benefits with self-test use and to explore the impact of self-testing for health-care use. Furthermore, the results will be used as an input for a follow-up study which is aimed at the development of consumer information, including a decision aid, for self-testing. By developing new consumer information for self-testing, we aim to provide consumers with a basis for a deliberate and informed choice.

### Theoretical framework

The Health Belief Model (HBM) [9] as well as the Theory of Planned Behaviour (TPB) [10] will be used as the theoretical framework in constructing the questionnaires (Study 1) and the interview topic guides (Study 2). The HBM was originally designed to explain health behaviours, such as screening, which may be comparable to self-testing. Nowadays the model has been used to explain and predict a wide variety of health-related behaviours (e.g. diabetes self-care, alcohol consumption, and smoking) [9,11,12]. The HBM is based on psychological expectancy-value models which state that human behaviour is the result of the valence that an individual relates to a specific goal and on the individual's assessment that a certain action will accomplish that goal. The HBM hypothesizes that health-related behaviour is the result of the individual's appraisal of the susceptibility to and the severity of a certain condition or illness and the individual's belief that a specific health action would result in a decrease of the susceptibility to or the severity of that condition. However, the health-related behaviour will only be performed if perceived barriers to that behaviour are compensated by the perceived benefits of performing it. Additionally, action is more likely to be initiated if cues (e.g. bodily or environmental events) are available and if the person has a high level of self-efficacy; e.g. if the person is confident that he or she is able to perform that action [9,11,13] .

Although the HBM and the TPB partially overlap, the TPB adds some important concepts to the HBM. According to the TPB [10], intention is the most proximal determinant of behaviour. Intention, in turn, is determined by three constructs which are conceptually independent: attitude, subjective norms, and perceived behavioural control. Attitude is based on an evaluation of the individual's positive and negative beliefs about consequences of that behaviour [10]. Subjective norms represent the idea that important others approve or disapprove of the person performing that behaviour. Perceived behavioural control is approximately similar to the self-efficacy concept of the HBM which comprises the individual's belief that (s)he is able to successfully perform the behaviour [9,12]. According to the TPB, perceived behavioural control influences behaviour both directly and indirectly, through intention [14]. The HBM as well as the TPB have shown to successfully explain several health-related behaviours [9,11,12,15-17].

### Study aim and research questions

The present study aims to validate the 2006 findings on frequency in self-test use, to describe information needs and information use of self-testers and non-testers, as well as the self-testers' further management on the basis of the test result. Furthermore, the quality of the existing consumer information for the most frequently used self-tests will be described.

This study will investigate the following research questions:

1. What is the frequency of self-test use?

2. What are the consumers' reasons for using or not using a self-test?

3. What information do self-testers use in the different self-test stages and what is the consumers' interpretation of the quality of this information?

4. What is the consumers' response to self-test results in terms of their confidence in the result, reassurance by the test result, and follow-up behaviour?

5. What information do consumers need in the decision making process of using a self-test or not using a self-test, and in further management on the basis of the self-test result?

6. What is the quality of the currently available consumer information on a selected set of self-tests?

## Methods

### Study design

Mixed methods study with (1) a cross-sectional study consisting of a two-phase Internet-questionnaire, (2) semi-structured interviews with self-testers and consumers who intend to perform a self-test, and (3) the assessment of the quality of consumer information accompanying self-tests.

### Ethical approval

The Medical Ethical Committee of Maastricht University indicated that no ethical approval was needed for this study.

### Study 1: Cross-sectional study

#### Aim

The aim of this cross-sectional study is to answer research questions (1) what is the frequency of self-test use?, (2) what are the consumers' reasons for using or not using a self-test?, (3) what information do self-testers use in the different self-test stages and what is the consumers' interpretation of the quality of this information?, and (4) what is the consumers' response to self-test results in terms of their confidence in the result, reassurance by the test result, and follow-up behaviour?

#### Selection criteria

Individuals aged 12 years or older who have an e-mail address will be randomly selected from the Flycatcher Internet-panel.

#### Methods of data collection

The survey will be conducted by Flycatcher, a Dutch ISO-certified institute for online research (ISO 26361 and ISO 20252), which will handle the recruitment of participants and the distribution of the questionnaires. Individuals aged 12 years or older who have an e-mail address can apply for the Flycatcher Internet-panel by registering at the Flycatcher website http://www.flycatcher.eu. New panellists are recruited through various channels: e.g. 'send-to-a-friend' actions by existing panel members, newsletters, directories of third parties (after permission), other private panels (after permission), and word-of-mouth advertising. Panellists are invited by e-mail to participate in a survey. This e-mail contains a short description of the study and an expiration date for filling out the survey. Panellists receive a gift voucher when they have completed a certain number of questionnaires.

In this survey, two consecutive questionnaires will be used. The first questionnaire will be based on the questionnaire that was used in the 2006 study to identify the use of self-tests [1]. This questionnaire will be sent to approximately 6,500 panellists and will determine the prevalence of and intention to use self-tests, types of self-tests used, and a number of demographic and lifestyle characteristics of participants (e.g. gender, age, level of education, perceived health status). The second questionnaire will be based on results of the 2006 study [1,18], and on consensus among the research team. This questionnaire will be sent to a selection of self-testers and will determine the information needs and information use, the consumers' interpretation of the quality of this information, the consumers' confidence in and their reassurance by the test-result, and their follow-up behaviour. Both questionnaires will be piloted in a small sample of the target population before being distributed on the Internet. The questionnaire will start with a short introduction of the purpose of the study and will provide a definition of self-testing with examples of what is considered a self-test and what is not. Finally, consent for recontacting respondents for further participation in the study will be asked. After one week, one reminder will be sent to non-responders. Both questionnaires are available as additional files 1 and 2.

#### Justification of sample size

Results of the 2006 study indicated that 16% of respondents had used a self-test. Based on Flycatcher's previous experiences we expect a response rate of 60%. The survey will be sent to 6500 respondents, which will result in a response of 3900 respondents and will include approximately 700 self-testers and 3200 non-testers.

#### Methods of data analysis

Analysis will be performed by using SPSS 16.0. Basic descriptive statistics will be used to describe the respondents' socio-demographic and lifestyle characteristics, the frequency of self-test use, their information use and information needs during the different self-test phases, the degree of consumer confidence in and reassurance by the test-result, and their follow-up behaviour.

### Study 2: Semi-structured interviews

#### Aim

The purpose of the semi-structured interviews will be to gain more in-depth understanding of consumers' considerations about self-testing. More specifically, we aim to answer research questions (2) what are the consumers' reasons for using or not using a self-test?, (3) what information do self-testers use in the different self-test stages and what is the consumers' interpretation of the quality of this information?, (4) what is the consumers' response to self-test results in terms of their confidence in the result, reassurance by the test result, and follow-up behaviour?, and (5) what information do consumers need in the decision making process of using a self-test or not using a self-test, and in further management on the basis of the self-test result?

#### Selection criteria

Individuals who indicate in the Internet survey that they are willing to be recontacted for participating in a face-to-face interview, will be approached. From this selection, individuals will be contacted if they performed a top-five most frequently used self-test within the last two years or intend to perform such a test, and live within a two-hour drive radius. We will purposively sample consumers with regard to gender, age, and level of education.

#### Methods of data collection

Semi-structured interviews will be held in order to explore the considerations of self-testers and consumers who intend to perform a self-test about using or potentially using a self-test. Respondents will be approached by e-mail that provides a short introduction of the research team and the research project, a description of the purpose of the interview, the duration of the interview and the incentive that participants will receive (€25 for a 1-hour interview). Furthermore, individuals will be asked to provide their name and phone number by e-mail if they are willing to participate in an interview. The researchers will contact them by phone to schedule an appointment for the interview. A semi-structured topic guide will be used for the interviews in which the reasons to use a self-test, the user-friendliness of the test, the interpretation and perceived reliability of the test result, the information needs and use, and follow-up actions based on the test result will be addressed. Interviews will be held at the homes of the participants. At least 8 participants per test will be interviewed and in case data-saturation is not met, additional participants will be invited for an interview. All interviews concerning a particular test will be held by one researcher (JG, MI, or a medical student) and will be audio-taped. The semi-structured topic guides for testers and consumers who intent to perform a self-test are available as additional files 3 and 4.

#### Justification of sample size

Assuming that 50% of respondents who have used a self-test and 25% of respondents who intend to use a self-test respond to the invitation to take part in an interview, we will need to approach 100 self-testers in total (20 persons per test divided over 5 tests) and 60 respondents who intend to use a self-test to take part in the interviews (30 persons per test divided over 2 tests).

#### Methods of data analysis

The audio taped interviews will be transcribed verbatim and imported as text documents into the qualitative analysis program, NVivo 2.0, that will be used to code and analyze the data. Analysis will be performed by using qualitative content analysis with a directed approach. Directed content analysis is aimed at validating or conceptually extending a theoretical framework. Existing theory serves as a basis for an initial coding scheme. Each code is accompanied by a specific definition which is derived from the theory [19,20]. Our coding scheme will reflect the concepts of the HBM [9,12] and the TPB [10]. The initial coding scheme will be developed by two researchers (JG and MI) and will cover the topics of the interview route (e.g. self-testing in general, reasons for testing, performing the self-test, information, and follow-up behaviour) and prompts (e.g. barriers, benefits, self-efficacy). Furthermore, free codes will be formulated to label additional topics that emerge during data-analysis.

Two researchers (JG and MI) will independently code all interviews. Meetings will be held in order to check the coding on exhaustiveness and appropriateness. Furthermore, it will be verified whether all theoretical concepts are identified and correctly categorized. Disagreements will be solved by comparing the text fragments and the conceptual definitions of the codes until consensus is reached. If consensus is not reached, a third independent rater will be appointed in order to achieve consensus (GR). Finally, all free codes will be integrated in the original coding scheme that is conceptually similar to the HBM [9,12] and TPB [10]. If these free codes appear to be a separate concept, the theoretical framework will be extended.

After coding is completed, the results for the different self-tests will be compared and assessed on their similarities. If certain self-tests are conceptually similar to one another, clusters will be made based on diseases and risk factors, (e.g. a cluster for CVD and related risk factors consisting of diabetes and cholesterol tests or a cluster for STDs consisting of Chlamydia and HIV). Analyses will result in a description of self-test behaviour, information needs and use of consumers, and their follow-up behaviour based on the test-result.

### Study 3: Assessment of the quality of consumer information of self-tests

#### Aim

The purpose of this study is to assess the quality of the instruction leaflets that are included in diagnostic self-test kits for home-use that are available on the Internet (research question 6).

#### Selection criteria

We will only include diagnostic self-tests for home use on body materials with the aim to diagnose a certain disease or risk factor. The home-tests under consideration will be the top-5 most frequently used self-tests in the Netherlands [1].

#### Methods of data collection

Dutch and English consumer information concerning the selected set of self-tests will be collected. First, we will perform an Internet search using Google which will only be aimed at websites which are available in Dutch or in English. If more than one producer has marketed one specific test, all available types will be included. The following key words will be used: selftesting, selftest, self test, home test, laboratory test, point-of-care test, direct-access test, lab test, home collect test. Additionally, we will search the Internet for specific tests and/or producers. The first 7 pages that come up at each key word will be consulted. If relevant links are identified in one of these pages, these sites will also be consulted. An overview will be made of the different test kits. The available consumer information will be downloaded from the Internet. Second, if the consumer information is not available on the Internet, producers or suppliers of self-tests will be contacted in order to collect this information.

#### Methods of data analysis

A checklist will be developed based on the validated English version of the International Patient Decision Aids Standard (IPDAS)[21,22], the DISCERN criteria [23,24], and the Dutch regulations for content of IVD patient information [25,26]. Additionally, items derived from the Internet survey and qualitative research will be included (e.g. which information is important according to consumers). The research team will be asked to assess the resulting initial and provisional checklist and to provide suggestions for improvement. Based on these comments, the checklist will be adapted and will be reviewed by the research team once again. If written comments are unclear, the specific member of the research team will be consulted for additional oral comments.

The checklist will be tested for its usability by applying the list on consumer information that was collected in the Internet search. Two researchers will each code three different leaflets of users' instructions. Each criterion will be coded on a 5-point Likert-scale which ranges from 1 = *definite no - the quality criterion has not been fulfilled at all*, to 5 = *definite yes - the quality criterion has been completely fulfilled*. The researchers have the opportunity to indicate if they perceived any problems when applying the checklist. Potential problems or suggestion for better use will be incorporated in the final version of the checklist.

Finally, the consumer information will be coded by means of the checklist. Furthermore, cut-off points will be determined to establish the overall quality level of the information. Two coders will independently code each leaflet of consumer information that was identified. Consensus meetings will be held in order to reach valid judgment of the quality of the consumer information.

## Discussion

Self-testing is a relatively new area and is still in a state of flux. Since the start of our 2006 study, we have observed a shift in the self-testing area. In 2006, most self-tests were at the consumer's own expense. In the last few years we observed that associations like the Dutch Kidney Association and the Municipal Health Services actively offer free self-tests to the Dutch public to detect kidney disease or Chlamydia infection. Media campaigns alert the Dutch public on the option to order a self-test for kidney disease or the target group receives an invitation by mail to order a self-test for Chlamydia. The tests are sent at no charge to the home of all individuals who request for it on a website [27,28]. We consider this a grey area in between pure self-diagnosis on the consumer's own initiative and testing initiated by a professional. Furthermore, some of the larger Dutch Health Insurance Companies partly compensate for the costs of a self-test. As a result of these developments in combination with the increase in Internet use and the current development of patient autonomy in health care, it is expected that self-tests are likely to become even more easily available and more widely used.

The studies described in this protocol are characterized by some innovative aspects. First, information about self-testing is scarce. Most studies on self-testing have focused on socio-demographic characteristics [1,2,5,8,29-32] of self-testers and technical aspects of self-tests [33-39]. To the best of our knowledge, studies concerning the considerations of individuals who intend to perform a self-test are lacking. In our opinion, this group is still undecided about performing or not performing a self-test and therefore has the highest information needs concerning self-testing. It is important to guide these individuals in making an informed choice about performing or not performing a self-test. Second, by combining quantitative and qualitative studies into a mixed-methods design, we aim to gain both general and more in-depth insight into considerations of self-testers and individuals who intend to perform a self-test.

By using an Internet research institute we are able to reach a large group of individuals which is necessary to gain insight into the general public's considerations about self-testing. On the one hand, it can be argued that using an Internet panel may result in a selection bias because these are individuals who are highly motivated to participate in surveys and who might be more concerned with their health. On the other hand, studies on Internet surveys versus paper-and-pencil surveys indicate that Internet surveys may yield similar results as traditional paper-and pencil surveys [40-42]. The Internet panel is not completely representative of the general Dutch population as panellists are slightly younger, more often female and more highly educated [1]. However, the Internet research institute claims that their panel is representative of the Dutch Internet population and since most self-tests are bought via the Internet, we consider the use of this panel as an acceptable option.

From a methodological perspective, a large scale Delphi study would have been optimal to develop the checklist assessing the quality of the consumer information about self-testing. However, due to financial and logistic restrictions, we use a small scale Delphi study consisting of experts of our research team. We believe that the checklist will have satisfying face-validity, is evidence-based, and is well-founded in theory since the checklist will be based on the results of our 2006 study, the results of studies one and two described in this protocol, and on the IPDAS criteria for decision aids [21,22], the Discern criteria for judging the quality of written consumer information on treatment choices [23,24], and the Dutch regulations for content of IVD patient information [25,26].

The study will allow us to get more insight into the considerations of self-testers and individuals who intend to perform a self-test and on the quality of the currently available consumer information. As far as we are aware, this will be the first study which focuses on these different aspects of self-testing simultaneously. The findings from the studies described above will provide the basis for new to develop consumer information on self-testing which will be tested on its effectiveness using an RCT. The development process and the outline of the RCT will be described in a second study protocol.

## Abbreviations

CVD: Cardiovascular diseases; HBM: Health Belief Model; IPDAS: International Patient Decision Aids Standard; ISO: International Organization for Standardization; IVD: In-Vitro Diagnostics; STD: Sexually transmitted diseases; TPB: Theory of Planned Behaviour; RCT: Randomized Controlled Trial

## Competing interests

The authors declare that they have no competing interests.

## Authors' contributions

JG is involved in the development of the questionnaires, interview schemes, and the checklist, as well as performing the analyses, the reporting aspects of the studies described in this protocol, and drafted the manuscript. MI is involved in the development of the questionnaires and the interview schemes, as well as the analyses and reporting aspects of the studies described in this protocol, and drafted the manuscript. JG and MI contributed equally substantially to the production of this manuscript. NdV and GJD are involved in critically revising the manuscript and provided valuable theoretical and design suggestions. GR and TvdW both conceived of the study, participated in its design and coordination, and helped to draft the manuscript. All authors have read and approved the final version of the manuscript.

## Funding

This study is financed by the Netherlands Organisation for Health Research and Development (ZonMW Prevention, subprogram Innovation), grant number 50-50101-96-406. Supplementary financial support has been provided by the Centraal Ziekenfonds (CZ) health insurance company. None of the sources of funding influenced either the study design, the writing of the manuscript, or the decision to submit the manuscript for publication.

## Pre-publication history

The pre-publication history for this paper can be accessed here:

http://www.biomedcentral.com/1471-2458/10/453/prepub

## Supplementary Material

Additional file 1 **Initial questionnaire cross sectional study**. This questionnaire is aimed to determine the prevalence of and intention to use self-tests, types of self-tests used, and a number of demographic and lifestyle characteristics of the participants. Click here for file

Additional file 2 **Second questionnaire cross sectional study**. This questionnaire is aimed at determining the information needs and information use, the consumers' interpretation of the quality of this information, the consumers' confidence in and their reassurance by the test-result, and their follow-up behaviour. Click here for file

Additional file 3 **Interview protocol self-testers**. Semi-structured topic guide used for the interviews which addresses the reasons to use a self-test, the user-friendliness of the test, the interpretation and perceived reliability of the test result, the information needs and use, and follow-up actions based on the test result. Click here for file

Additional file 4 **Interview protocol consumers who intend to use a self-test**. Semi-structured topic guide used for the interviews which addresses the reasons to use a self-test, the user-friendliness of the test, the interpretation and perceived reliability of the test result, the information needs and use, and follow-up actions based on the test result. Click here for file

## References

[B1] Ronda G Portegijs P Dinant GJ Buntinx F Norg R Van der Weijden T Use of diagnostic self-tests on body materials among Internet users in the Netherlands: prevalence and correlates of use BMC Public Health 2009 9 100 10.1186/1471-2458-9-100 19358708 PMC2675528

[B2] Deutekom M Aziz Y van Dis I Stronks K Bossuyt PM De Nationale Cholesteroltest: vooral gezonde deelnemers [The Dutch National Cholesterol Test: participants mainly healthy] Nederlands Tijdschrift voor Geneeskunde 2008 152 2425 2429 19055144

[B3] Ryan A Greenfield S Wilson S Prevalence and determinants of the use of self-tests by members of the public: a mixed methods study BMC Public Health 2006 6 193 10.1186/1471-2458-6-193 16869960 PMC1562406

[B4] Ryan A Wilson S Greenfield S Clifford S McManus RJ Pattison HM Range of self-tests available to buy in the United Kingdom: an Internet survey Journal of Public Health 2006 28 370 10.1093/pubmed/fdl051 17052990

[B5] Colfax GN Lehman JS Bindman AB Vittinghoff E Vranizan K Fleming PL Chesney M Osmond D Hecht FM What happened to home HIV test collection kits? Intent to use kits, actual use, and barriers to use among persons at risk for HIV infection Aids Care 2002 14 675 682 10.1080/0954012021000005533a 12419117

[B6] Campbell S Klein R Home Testing To Detect Human Immunodeficiency Virus: Boon or Bane? Journal of Clinical Microbiology 2006 44 3473 3476 10.1128/JCM.01511-06 17021069 PMC1594804

[B7] Frith L HIV self-testing: a time to revise current policy Lancet 2007 369 243 245 10.1016/S0140-6736(07)60113-5 17240291

[B8] Phillips KA Chen JL Willingness to use instant home HIV tests Data from the California Behavioral Risk Factor Surveillance Survey American Journal of Preventive Medicine 2003 24 340 348 10.1016/S0749-3797(03)00019-9 12726872

[B9] Janz NK Champion VL Strecher VJ Glanz K, Rimer BK, Lewis FM The Health Belief Model Health behavior and health education: Theory, research, and practice 2002 3 San Francisco: Jossey-Bass 45 66

[B10] Ajzen I The theory of planned behavior Organizational Behavior and Human Decision Processes 1991 50 179 211 10.1016/0749-5978(91)90020-T

[B11] Bartolomew LK Parcel GS Kok G Gottlieb NH Bartolomew LK, Parcel GS, Kok G, Gottlieb NH Behavior-oriented theories used in health promotion Planning health promotion programs: An Intervention Mapping approach 2006 1 San Francisco, CA: Jossey-Bass

[B12] Janz NK Becker MH The Health Belief Model: A Decade Later Health Education Quarterly 1984 11 1 6392204 10.1177/109019818401100101

[B13] Rosenstock IM Strecher VJ Becker MH DiClemente RJ, Peterson JL The health belief model and HIV risk behavior change Preventing Aids: Theories and methods of behavioral interventions 1994 New York, NY: Springer 5 24

[B14] Kok G de Vries H Mudde AN Strecher VJ Planned health education and the role of self-efficacy: Dutch research Health Education Research 1991 6 231 238 10.1093/her/6.2.231

[B15] Armitage CJ Conner M Efficacy of the Theory of Planned Behaviour: A meta-analytic review British Journal of Social Psychology 2001 40 471 499 10.1348/014466601164939 11795063

[B16] Conner M Armitage CJ Extending the theory of planned behavior: A review and avenues for further research Journal of Applied Social Psychology 1998 28 1429 1464 10.1111/j.1559-1816.1998.tb01685.x

[B17] Cooke R French DP How well do the theory of reasoned action and theory of planned behaviour predict intentions and attendance at screening programmes? A meta-analysis Psychology and Health 2008 23 745 765 10.1080/08870440701544437 25160879

[B18] CVZ-rapport Diagnostische selftests op lichaamsmateriaal. Aanbod, validiteit en gebruik door de consument [Diagnostic self-tests on bodymaterial. Supply, validity, and use by the consumer] 2007

[B19] Harting J Rutten GMJ Rutten STJ Kremers SPJ A qualitative application of the Diffusion of Innovations Theory to examine determinants of guideline adherence among physical therapists Physical Therapy 2009 89 221 232 19168713 10.2522/ptj.20080185

[B20] Hsieh H Shannon SE Three approaches to qualitative content analysis Qualitative Health Research 2005 15 1277 1288 10.1177/1049732305276687 16204405

[B21] Elwyn G O'Connor A Stacey D Volk R Edwards A Coulter A Thomson R Barratt A Barry M Bernstein S Developing a quality criteria framework for patient decision aids: online international Delphi consensus process British Medical Journal 2006 333 417 423 10.1136/bmj.38926.629329.AE 16908462 PMC1553508

[B22] Elwyn G O'Connor AM Bennett C Newcombe RG Politi M Durand MA Drake E Joseph-Williams N Khangura S Saarimaki A Assessing the quality of decision support technologies using the International Patient Decision Aid Standards instrument (IPDASi) PlosOne 2009 4 10.1371/journal.pone.0004705 PMC2649534 19259269

[B23] Charnock D Shepperd S Needham G Gann R DISCERN: an instrument for judging the quality of written consumer health information on treatment choices Journal of Epidemiology and Community Health 1999 53 105 111 10.1136/jech.53.2.105 10396471 PMC1756830

[B24] Discern Genetics Quality Criteria http://www.discern-genetics.org/

[B25] Health Council of the Netherlands Annual report on screening for disease 2007 - The self-testing of body samples 2007 The Hague: Health Council of the Netherlands

[B26] Wielders JPM Loeber JG Bartels PCM Praktische consequenties van IVD-Richtlijn voor laboratoria, kansen en beperkingen Nederlands Tijdschrift voor Klinische Chemie en Labgeneeskunde 2004 29 209 213

[B27] Nielen M Schellevis F Verheij R The usefulness of a free self-test for screening albuminuria in the general population: a cross-sectional survey BMC Public Health 2009 9 381 10.1186/1471-2458-9-381 19818129 PMC2767353

[B28] Op de Coul E Weenen T Van der Sande M Van den Broek I Process evaluation of the Chlamydia Screening Implementation in the Netherlands: phase 1. Challenges and opportunities during preparation and first operational phase The National Institute for Public Health and the Environment 2009

[B29] Ryan A Wilson S Taylor A Greenfield S Factors associated with self-care activities among adults in the United Kingdom: a systematic review BMC Public Health 2009 9 96 10.1186/1471-2458-9-96 19344526 PMC2674604

[B30] Shiloh S Vinter M Barak M Correlates of health screening utilization: The roles of health beliefs and self-regulation motivation Psychology and Health 1997 12 301 317 10.1080/08870449708406709

[B31] Ford CA Jaccard J Millstein SG Viadro CI Eaton JL Miller WC Young adults' attitudes, beliefs, and feelings about testing for curable STDs outside of clinic settings Journal of Adolescent Health 2004 34 266 269 15040995 10.1016/j.jadohealth.2003.07.013

[B32] Skolnik HS Phillips KA Binson D Dilley JW Deciding where and how to be tested for HIV: what matters most? Journal of Acquired Immune Deficiency Syndrome 2001 27 292 10.1097/00126334-200107010-00013 11464151

[B33] Bais AG van Kemenade FJ Berkhof J Verheijen RH Snijders PJ Voorhorst F Babovic M van Ballegooijen M Helmerhorst TJ Meijer CJ Human papillomavirus testing on self-sampled cervicovaginal brushes: an effective alternative to protect nonresponders in cervical screening programs International Journal of Cancer 2007 120 1505 1510 10.1002/ijc.22484 17205514

[B34] Belinson JL Pretorius RG Enerson C Garcia F Cruz EP Belinson SE Yeverino Garcia E Brainard J The Mexican Cervical Cancer Screening Trial: self-sampling for human papillomavirus with unaided visual inspection as a secondary screen International Journal of Gyneocological Cancer 2009 19 27 32 10.1111/IGC.0b013e318197f479 19258937

[B35] Hsieh YH Howell MR Gaydos JC McKee KT Jr Quinn TC Gaydos CA Preference among female Army recruits for use of self-administrated vaginal swabs or urine to screen for Chlamydia trachomatis genital infections Sexually Transmitted Diseases 2003 30 769 773 10.1097/01.OLQ.0000079048.11771.46 14520176

[B36] Kahn JA Slap GB Huang B Rosenthal SL Wanchick AM Kollar LM Hillard PA Witte D Groen P Bernstein DI Comparison of adolescent and young adult self-collected and clinician-collected samples for human papillomavirus Obstetrics and Gynecology 2004 103 952 959 15121570 10.1097/01.AOG.0000124569.61462.8d

[B37] Nielen MM Schellevis FG Verheij RA The usefulness of a free self-test for screening albuminuria in the general population: a cross-sectional survey BMC Public Health 2009 9 381 10.1186/1471-2458-9-381 19818129 PMC2767353

[B38] Szarewski A Cadman L Mallett S Austin J Londesborough P Waller J Wardle J Altman DG Cuzick J Human papillomavirus testing by self-sampling: assessment of accuracy in an unsupervised clinical setting Journal of Medical Screening 2007 14 34 42 10.1258/096914107780154486 17362570 PMC4109399

[B39] Mahilum-Tapay L Laitila V Wawrzyniak JJ Lee HH Alexander S Ison C Swain A Barber P Ushiro-Lumb I Goh BT New point of care Chlamydia Rapid Test bridging the gap between diagnosis and treatment: performance evaluation study British Medical Journal 2007 335 1190 10.1136/bmj.39402.463854.AE 18055487 PMC2128659

[B40] Donovan MA Drasgow F Probst TM Does computerizing paper-and-pencil job attitude scales make a difference? New IRT analyses offer insight Journal of Applied Psychology 2000 85 305 313 10.1037/0021-9010.85.2.305 10783546

[B41] Knapp H Kirk SA Using pencil and paper, Internet and touch-tone phones for self-administered surveys: does methodology matter? Computers in Human Behavior 2003 19 117 134 10.1016/S0747-5632(02)00008-0

[B42] Mead AD Drasgow F Equivalence of computerized and paper-and-pencil cognitive ability tests: A meta-analysis Psychological Bulletin 1993 114 449 449 10.1037/0033-2909.114.3.449

