# Supplementary material for: Improving behaviour in self-testing (IBIS): Study on frequency of use, consequences, information needs and use, and quality of currently available consumer information (protocol)
Source: BMC Public Health. 2010 Aug 3;10:453. doi: 10.1186/1471-2458-10-453 (PMC2919481; doi:10.1186/1471-2458-10-453)
Supplement: Additional file 2 — Second questionnaire cross sectional study. This questionnaire is aimed at determining the information needs and information use, the consumers' interpretation of the quality of this information, the consumers' confidence in and their reassurance by the test-result, and their follow-up behaviour. [file 1471-2458-10-453-S2.DOC]

**Second questionnaire cross sectional study**

**The questionnaire was originally in Dutch and has been translated into English**

Some time ago, you indicated on a questionnaire that you had used a …[dynamic representation] self-test (*indicate form*). The questions below are about this self-test and various aspects associated with it.

The first questions ask you for general information on the self-test you did. This is followed by a number of questions about the various phases of the self-testing process:

1. before you take the test;
2. the actual use of the test (only for tests done at home);
3. interpreting the test result;
4. what to do with the test result.

The heading at the top of each page indicates what phase of the process the questions are about.

#### **Questions specifically about the** [dynamic representation] **self-test**

**1a. In what form did you take the [dynamic respresentation] self-test?**

- I did the self-test myself, at home.
- I went to a particular place (supermarket, pharmacy, chemist’s), had the test done there and was given the results immediately.
- I went to a laboratory, where they took some body materials, and I then received the results at home by mail, e-mail or through the internet.
- I sent off body materials to a laboratory and received the results at home by mail, e-mail or through the internet.

**1b. When did you take this self-test?**

- - - 0-3 months ago
    - 4- 6 months ago
    - 7-12 months ago
    - Over 12 months ago

**1c. What was the main reason for you to do a [dynamic representation] self-test?**

- - - Because I had a medical complaint.
    - Because I was worried that I might have a disease.
    - Because other people advised me to take the test.
    - Because people in my immediate environment had the disease.
    - Because I had had the test before and wanted to know if anything had changed.
    - Because I wanted to know more about my health status.
    - Other, namely ………………………………..

**1d. Why did you decide to take a self-test?** *(multiple answers allowed)*

- - - Because such a test ensures privacy.
    - Because you get the results quickly.
    - Because the test was offered to me (free of charge).
    - Because I wanted to take responsibility for my own health.
    - Because I previously asked my doctor to do the test but it was never done.
    - Other, namely…………………………………………………..

**1e. What was the result of the self-test?**

- Normal (nothing wrong)
- Abnormal (something wrong)
- Inconclusive
- Test failed
- Can’t remember

**The following questions are about the information you heard or read before you decided to do the self-test (or have it done)**

**2a. Before you tested yourself (or had yourself tested), had you heard or read any information about this test?**

- Yes, I had heard some information (proceed to 2c).
- Yes, I had read some information (proceed to 2b).
- Yes, I had heard and read some information (proceed to 2b).
- No (proceed to 2h).

**2b. What part of the information did you read?**

- I read all of the information.
- I looked at the headings and only read the parts that were interesting and/or relevant to me.
- I only read the beginning and/or the end of the information.
- I read some sentences here and there in the text to decide if the rest of it was interesting and/or relevant to me.

**2c. How did you get the information?**

- I asked for it myself or looked it up (proceed to 2d)
- Through others (proceed to 2e)
- I came upon it by accident (proceed to 2e)
- Other, namely ……….. (proceed to 2e)

**2d. Were you able to find the information you were looking for?**

- Yes
- No

**2e. Where or from whom did you get this information? (multiple answers allowed)**

- From relatives, friends or colleagues
- From my family doctor, from a specialist
- At my family doctor’s office (e.g. from a brochure)
- At the pharmacy
- At the chemist’s
- From TV or radio
- From newspapers, magazines or books
- From the Internet
- At the supermarket
- Other, namely…..

**2f. What was the information about? (multiple answers allowed)**

- For whom the test is intended
- How the test should be carried out
- What disease or risk factor the test identifies
- The reliability of the test result
- Where the test is done or sold
- The meaning of the test result
- The costs of the test, and if they are reimbursed by my insurance
- Whether I can have the test done at my family doctor’s
- Other, namely…….

**2g. What was your general opinion about the information you heard or read**?

| 1 Very poor | 2 | 3 | 4 | 5 very good |
| --- | --- | --- | --- | --- |
| 1 very hard to understand | 2 | 3 | 4 | 5 very easy to understand |
| 1 Very unclear | 2 | 3 | 4 | 5 very clear |
| 1 very unrelaible | 2 | 3 | 4 | 5 very reliable |
| 1 Very incomplete | 2 | 3 | 4 | 5 very complete |
| 1 Very difficult | 2 | 3 | 4 | 5 very easy |

**2h. In what way would you prefer to get information about self-tests? (multiple answers allowed)**

- From relatives, friends or colleagues
- From my family doctor, from a specialist
- At my family doctor’s office (e.g. from a brochure)
- At the pharmacy
- At the chemist’s
- From TV or radio
- From newspapers, magazines or books
- From the Internet
- At the supermarket
- Other, namely…...

**2i. What would be important for you to know before you do the test? (multiple answers allowed)**

- For whom the test is intended
- How the test should be carried out
- What disease or risk factor the test identifies
- The reliability of the test result
- Where the test is done or sold
- The meaning of the test result
- The costs of the test, and if they are reimbursed by my insurance
- Whether I can have the test done at my family doctor’s
- Other, namely…….

**The following questions are only intended for people who have done tests at home**

**The questions below are about the information you have used or read about the way you actually have to perform the test.**

**Some questions refer to the patient information leaflet; by this we mean the instructions for use included in the packaging of the test.**

**3a. Did you read the information on the packaging before you bought the test?**

- - Yes, all of it
  - Yes, part of it
  - No

**3b. Did you read (or re-read) the information on the packaging before you did the test?**

- - Yes, all of it
  - Yes, part of it
  - No

**If the answer to 3a AND 3b was ‘No’, proceed to question 3f; otherwise 3c**

**3c. Do you think the information on the packaging helped you to better perform the test?**

- Yes
- No

**3d. Open question: could you indicate what information you thought was missing from the packaging?...................................................................................................**

**3e. What was your general opinion of the information on the packaging**?

| 1 Very poor | 2 | 3 | 4 | 5 Very good |
| --- | --- | --- | --- | --- |
| 1 Very hard to understand | 2 | 3 | 4 | 5 Very easy to understand |
| 1 Very unclear | 2 | 3 | 4 | 5 Very clear |
| 1 Very unreliable | 2 | 3 | 4 | 5 Very reliable |
| 1 Very incomplete | 2 | 3 | 4 | 5 Very complete |
| 1 Very difficult | 2 | 3 | 4 | 5 Very easy |

**3f. Did you read the information in the patient information leaflet about the way to perform this self-test?**

- Yes, all of it
- Yes, part of it
- No (proceed to 3j)

**3g. In your opinion, did the information in the patient information leaflet help you to better perform the test?**

- Yes
- No

**3h. Open question: Could you indicate what information about performing the test was missing from the patient information leaflet?.......................................................................**

**3i. What was your general opinion of the information on performing the test that was given in the patient information leaflet**?

| 1 Very poor | 2 | 3 | 4 | 5 Very good |
| --- | --- | --- | --- | --- |
| 1 Very hard to understand | 2 | 3 | 4 | 5 Very easy to understand |
| 1 Very unclear | 2 | 3 | 4 | 5 Very clear |
| 1 Very unreliable | 2 | 3 | 4 | 5 Very reliable |
| 1 Very incomplete | 2 | 3 | 4 | 5 Very complete |
| 1 Very difficult | 2 | 3 | 4 | 5 Very easy |

**3j. Did you look for or get any other information about the way to perform the test besides the information on the packaging and/or in the patient information leaflet?**

- Yes, I looked for it (proceed to 3k)
- Yes, I got it (proceed to 3l)
- No (proceed to the questions about performing the test)

**3k. Did you actually find the information you were looking for?**

- Yes
- No

**3l. Where or from whom did you seek this additional information and where or from whom did you get it?**

- From relatives, friends or colleagues
- From my family doctor, from a specialist
- At my family doctor’s office (e.g. from a brochure)
- At the pharmacy
- At the chemist’s
- From TV or radio
- From newspapers, magazines or books
- From the Internet
- At the supermarket
- Other, namely…...

**3m. Do you think this additional information helped you to better perform the test?**

- Yes
- No, everything was already in the patient information leaflet
- No, it did not help me
- Other, namely……………………………….

**3n. Open question: Could you indicate what information about performing the test was missing from the additional information you got?...............................................................**

**The following questions are on the information you used or read about how to interpret the test result, in other words, about the meaning of the test result.**

**4a1. For tests used at home: were you able to see the test result clearly?**

- Yes
- No

**4a2. For tests used at home: did you read the information about the meaning of your test result in the patient information leaflet?**

- Yes (proceed to 4c)
- No (proceed to 4f)

**4a3. For lab tests and street-corner tests: were you given information about the meaning of your test result when you received the result?**

- Yes (proceed to 4b)
- No (proceed to 4f)

**4b. Did you read this information?**

- Yes, all of it
- Yes, part of it
- The information was given to me orally
- No

**4c. What was your general opinion on the information about the meaning of your test result**?

| 1 Very poor | 2 | 3 | 4 | 5 Very good |
| --- | --- | --- | --- | --- |
| 1 Very hard to understand | 2 | 3 | 4 | 5 Very easy to understand |
| 1 Very unclear | 2 | 3 | 4 | 5 Very clear |
| 1 Very unreliable | 2 | 3 | 4 | 5 Very reliable |
| 1 Very incomplete | 2 | 3 | 4 | 5 Very complete |
| 1 Very difficult | 2 | 3 | 4 | 5 Very easy |

**4d. Do you think this information helped you to better interpret your test result?**

- Yes
- No

**4e. Open question: Could you indicate what information about interpreting your test result was missing?..................................................................................**

**4f. Did you look for or get any additional information about interpreting your test result?**

- Yes, I looked for it (proceed to 4g)
- Yes, I received it (proceed to 4h)
  - No (please continue with question 5)

**4g. Did you actually find the additional information you were looking for?**

- Yes
- No

**4h. Where or with whom did you look for this additional information and where or from whom did you get it?**

- From relatives, friends or colleagues
- From my family doctor, from a specialist
- At my family doctor’s office (e.g. from a brochure)
- At the pharmacy
- At the chemist’s
- From TV or radio
- From newspapers, magazines or books
- From the Internet
- At the supermarket
- Other, namely…...

.

**4i. Do you think this additional information helped you to better interpret your test result?**

- Yes
- No, everything was already in the patient information leaflet
- No, it did not help me
- Other, namely……………………………….

**4j. Open question: Could you indicate what additional information you would have liked to have to better interpret your test result but couldn’t get?...........................................................**

**4k. What was your general opinion on the additional information about interpreting your test result**?

| 1 Very poor | 2 | 3 | 4 | 5 Very good |
| --- | --- | --- | --- | --- |
| 1 Very hard to understand | 2 | 3 | 4 | 5 Very easy to understand |
| 1 Very unclear | 2 | 3 | 4 | 5 Very clear |
| 1 Very unreliable | 2 | 3 | 4 | 5 Very reliable |
| 1 Very incomplete | 2 | 3 | 4 | 5 Very complete |
| 1 Very difficult | 2 | 3 | 4 | 5 Very easy |

**The following questions are about the results of this self-test.**

**5. To what extent do you agree with the following statements about the self-test?**

| **Statement** | **Completely disagree** | **Disagree** | **Neither agree nor disagree** | **Agree** | **Completely agree** |
| --- | --- | --- | --- | --- | --- |
| I have confidence in the results of this self-test. |  |  |  |  |  |
| If the test result is normal (nothing is wrong) you can assume that this test result is reliable. |  |  |  |  |  |
| If the test result is abnormal (something is wrong), you can assume that this result is reliable. |  |  |  |  |  |
| I have just as much confidence in this self-test as in a test ordered by a doctor. |  |  |  |  |  |
| I would recommend this self-test to others. |  |  |  |  |  |
| This self-test offers me certainty about my own health. |  |  |  |  |  |

**The following questions are about your test result and the follow-up steps you may have taken as a consequence of the result.**

**If the test result was normal (nothing wrong)**

**6. Did you have any medical complaints when you did this self-test?** *(loop: question is skipped if complaints were reason to take self-test (1c))*

- - Yes
  - No

**7. Did you repeat the test to make sure?**

- - - Yes
    - No

**8. Were you reassured by the result of this self-test?**

- - Yes, completely
  - Yes, partly
  - No

**9. What did you do after you got the test result?** *(multiple answers allowed)*

- - - I took no further action.
    - I discussed the test result with relatives or friends.
    - I looked for further information.
    - I did a self-test for other diseases or risk factors.
    - I changed my lifestyle.
    - I bought over-the-counter drugs (such as vitamins or pills).
    - I sought help from complementary medicine (such as acupuncture or homeopathy).
    - I consulted a doctor.
    - I consulted another care provider (such as a dietician, a psychologist, a physiotherapist, my GP’s receptionist or a nurse practitioner).
    - Other

**9a. *If looked for further information:* Where did you look for further information?**

- At my family doctor’s office (e.g. from a brochure)
- At the pharmacy
- At the chemist’s
- From TV or radio
- From newspapers, magazines or books
- From the Internet
- At the supermarket
  - Other

**9b. *If lifestyle changed:* What lifestyle changes did you make? *(multiple answers allowed*)**

- - - Took some rest
    - Avoided stress / took things a bit easier
    - Started to eat healthier food / changed my diet
    - Used less alcohol
    - Gave up smoking
    - Took more exercise
    - Had safe sex
    - Bought over-the-counter drugs
    - Avoided things I’m allergic to
    - Other

**9c. *If doctor was consulted:* What kind of doctor did you consult?**

- - A general practitioner
  - A specialist at the hospital
  - A doctor at the regional public health service
  - Other

*Next questions only if a doctor was consulted*

**10. What was your main reason for consulting a doctor?**

- - I wanted to discuss my medical complaint with the doctor.
  - I wanted to discuss my concern about having a particular disease or health risk.
  - I wanted to ask for further information about the test.
  - I wanted to ask for further information about the disease for which I used the test.
  - I wanted to discuss my test result with a doctor.
  - I wanted the doctor to repeat the test.
  - I wanted the doctor to do other tests.
  - I wanted to be referred to hospital.

**10a *If doctor was asked to repeat the test:* Why did you want the test to be repeated by the doctor?**

- - I did not trust the result of the self-test.
  - I wanted more certainty about the result of the self-test.
  - I was not sure if I had done the test correctly.
  - I was not sure if this self-test was the right one to find out about the disease or the risk.

**11. Did you tell the doctor you had done a self-test?**

- - - Yes
    - No

**11a. *If answer to question 11 was Yes:* Did the doctor explain to you what the result of the self-test meant for your situation?**

- - Yes
  - No

**12. Did the doctor repeat the same test you did as a self-test, or order the same test to be done, for instance at the hospital?**

- - Yes
  - No

**12a. *If answer to question 12 was Yews:* Was the result of your self-test the same as that of the test done or ordered by the doctor?**

- - - Yes
    - No

**13. Did the doctor order any other tests?**

- - Yes
  - No

**14. Did the doctor reassure you?**

- - - Yes, completely
    - Yes, partly
    - No

**15. Were you satisfied with what the doctor did or told you?**

- - Yes, completely satisfied
  - Yes, partly satisfied
  - Neither satisfied nor dissatisfied
  - No partly dissatisfied
  - No, completely dissatisfied

**16. Did the doctor give you any treatment because of the result of the self-test?**

- - - Yes, he gave me treatment for the disorder I had tested myself for
    - Yes, but for a different disorder
    - No

**17. Did the doctor refer you to hospital as because of the results of the self-test?**

- - Yes, for the disorder I had tested myself for
  - Yes, but for a different disorder
  - No

*For all respondents:*

**18. Are you currently being treated for the disorder for which you tested yourself?**

- - Yes
  - No

**If test result was abnormal (something wrong)**

**6. Did you have any medical complaints when you did the self-test?** *(loop: question is skipped if complaints were reason to take self-test)*

- - Yes
  - No

**7. Did you repeat the test to make sure?**

- - - Yes
    - No

**8. What did you do after you got the test result? (Multiple answers allowed)**

- - - I took no further action.
    - I discussed the test result with relatives or friends.
    - I looked for further information.
    - I did a self-test for other diseases or risk factors.
    - I changed my lifestyle.
    - I bought over-the-counter drugs (such as vitamins or pills).
    - I sought help from complementary medicine (such as acupuncture or homeopathy).
    - I consulted a doctor.
    - I consulted another care provider (such as a dietician, a psychologist, a physiotherapist, my GP’s receptionist or a nurse practitioner).
  - Other

**8a. *If no further action was taken:* Why did you take no further action?**

- - - I already knew about the disease or the risk.
    - I did not trust the test result.
    - I did not know what to change / was unable to change anything.

***8b If further information was sought:* Where did you look for further information?**

- At my family doctor’s office (e.g. from a brochure)
- At the pharmacy
- At the chemist’s
- From TV or radio
- From newspapers, magazines or books
- From the Internet
- At the supermarket
  - Other

**8c *If lifestyle was changed:* What lifestyle changes did you make? *(Multiple answers allowed)***

- - - Took some rest
    - Avoided stress / took things a bit easier
    - Started to eat healthier food / changed my diet
    - Used less alcohol
    - Gave up smoking
    - Took more exercise
    - Had safe sex
    - Bought over-the-counter drugs
    - Avoided things I’m allergic to
  - Other

**8d *If doctor was consulted:* What kind of doctor did you consult?**

- - A general practitioner
  - A specialist at the hospital
  - A doctor at the regional public health service
    - Other

*Next questions only if a doctor was consulted*

**9. What was your main reason for consulting a doctor?**

- - I wanted to discuss my medical complaint with the doctor.
  - I wanted to discuss my concern about having a particular disease or health risk.
  - I wanted to ask for further information about the test.
  - I wanted to ask for further information about the disease for which I used the test.
  - I wanted to discuss my test result with a doctor.
  - I wanted the doctor to repeat the test.
  - I wanted the doctor to do other tests.
  - I wanted to get treatment for the disease or health risk I had discovered by means of the test.
  - I wanted to be referred to hospital.

**9a *If doctor was asked to repeat the test:* Why did you want the test to be repeated by the doctor?**

- - I did not trust the result of the self-test.
  - I wanted more certainty about the result of the self-test.
  - I was not sure if I had done the test correctly.
    - I was not sure if this self-test was the right one to find out about the disease or the risk.

**10. Did you tell the doctor you had done a self-test?**

- - Yes
  - No

**10a *If answered to question 10 was Yes:* Did the doctor explain to you what the result of the self-test meant for your situation?**

- - - Yes
    - No

**11. Did the doctor repeat the same test you did as a self-test, or order the same test to be done, for instance at the hospital?**

- - Yes
  - No

***11a. If answer to question 11 was Yes:* Was the result of your self-test the same as that of the test done or ordered by the doctor?**

- - - Yes
    - No

**12. Did the doctor order any other tests?**

- - Yes
  - No

**13. Did the doctor reassure you?**

- - - Yes, completely
    - Yes, partly
    - No

**14. Were you satisfied with what the doctor did or told you?**

- - Yes, completely satisfied
  - Yes, partly satisfied
  - Neither satisfied nor dissatisfied
  - No, partly dissatisfied
  - No, completely dissatisfied

**15. Did the doctor give you any treatment because of the result of the self-test?**

- - - Yes, he gave me treatment for the disorder I had tested myself for
    - Yes, but for a different disorder
    - No

**16. Did the doctor refer you to hospital as because of the results of the self-test?**

- - Yes, for the disorder I had tested myself for
  - Yes, but for a different disorder
  - No

*For all respondents:*

**17. Are you currently being treated for the disorder for which you tested yourself?**

- - Yes
  - No

**If test failed / inconclusive / can’t remember**

**6. Did you have any medical complaints when you did the self-test?** *(loop: question is skipped if complaints were reason to take self-test)*

- - Yes
  - No

**7. Did you repeat the test to make sure?**

- - - Yes
    - No

**8. What did you do after you got the test result? (Multiple answers allowed)**

- - - I took no further action.
    - I discussed the test result with relatives or friends.
    - I looked for further information.
    - I did a self-test for other diseases or risk factors.
    - I changed my lifestyle.
    - I bought over-the-counter drugs (such as vitamins or pills).
    - I sought help from complementary medicine (such as acupuncture or homeopathy).
    - I consulted a doctor.
    - I consulted another care provider (such as a dietician, a psychologist, a physiotherapist, my GP’s receptionist or a nurse practitioner).
  - Other

**8a. *If further information was sought:* Where did you look for further information?**

- At my family doctor’s office (e.g. from a brochure)
- At the pharmacy
- At the chemist’s
- From TV or radio
- From newspapers, magazines or books
- From the Internet
- At the supermarket
  - Other

**8b. *If lifestyle was changed:* What lifestyle changes did you make? *(Multiple answers allowed)***

- - - Took some rest
    - Avoided stress / took things a bit easier
    - Started to eat healthier food / changed my diet
    - Used less alcohol
    - Gave up smoking
    - Took more exercise
    - Had safe sex
    - Bought over-the-counter drugs
    - Avoided things I’m allergic to
  - Other

**8c. *If doctor was consulted:* What kind of doctor did you consult?**

- - A general practitioner
  - A specialist at the hospital
  - A doctor at the regional public health service
    - Other

*Next questions only if a doctor was consulted*

**9. What was your main reason for consulting a doctor?**

- - Because the test had failed or the result was unclear.
  - I wanted to discuss my medical complaints with the doctor.
  - I wanted to discuss my concern about having a particular disease or health risk.
  - I wanted to ask for further information about the test.
  - I wanted to ask for further information about the disease for which I used the test.
  - I wanted to discuss my test result with a doctor.
  - I wanted a doctor to repeat the test.
  - I wanted a doctor to do other tests.
  - I wanted to be referred to hospital.

**10. Did you tell the doctor you had done a self-test?**

- - Yes
  - No

**11. Did the doctor repeat the same test you did as a self-test, or order the same test to be done, for instance at the hospital?**

- - Yes
  - No

**12. Did the doctor order any other tests?**

- - Yes
  - No

**13. Did the doctor reassure you?**

- - - Yes, completely
    - Yes, partly
    - No

**14. Were you satisfied with what the doctor did or told you?**

- - Yes, completely satisfied
  - Yes, partly satisfied
  - Neither satisfied nor dissatisfied
  - No, partly dissatisfied
  - No, completely dissatisfied

**15. Did the doctor give you any treatment because of the result of the self-test?**

- - - Yes, he gave me treatment for the disorder I had tested myself for
    - Yes, but for a different disorder
    - No

**16. Did the doctor refer you to hospital as because of the results of the self-test?**

- - Yes, for the disorder I had tested myself for
  - Yes, but for a different disorder
  - No

*For all respondents:*

**17. Are you currently being treated for the disorder for which you tested yourself?**

- - Yes
  - No
