# Supplementary material for: Improving behaviour in self-testing (IBIS): Study on frequency of use, consequences, information needs and use, and quality of currently available consumer information (protocol)
Source: BMC Public Health. 2010 Aug 3;10:453. doi: 10.1186/1471-2458-10-453 (PMC2919481; doi:10.1186/1471-2458-10-453)
Supplement: Additional file 3 — Interview protocol self-testers. Semi-structured topic guide used for the interviews which addresses the reasons to use a self-test, the user-friendliness of the test, the interpretation and perceived reliability of the test result, the information needs and use, and follow-up actions based on the test result. [file 1471-2458-10-453-S3.DOC]

**Interview protocol self-testers**

First of all I’d like to thank you very much for your willingness to take part in this interview. Let me first explain to you what this study is about. I work at Maastricht University and I’m doing a research study on self-tests. The aim of this study is to examine the experiences of people who have used self-tests. The ultimate goal of the study is to develop better information for a number of self-tests, including the cholesterol test.

Last autumn, you received and filled out two questionnaires about self-tests from Flycatcher, and you indicated then that you had done a [disease or risk factor] self-test.

I would like to talk with you about this test. We will discuss four aspects: why you decided to do a self-test, how you did the test, what you did after the test and what you thought about the information you were given about the test.

What matters in this interview is your experiences and opinions about this self-test. This means there are no “correct” or “incorrect” answers: I want to hear your opinion. Of course what you say in this interview will be treated confidentially. If I ask any questions that you’d rather not answer, please tell me so. With your permission I’s like to tape this interview. All your information will be processed anonymously and then destroyed, so nobody could trace it back to you.

The interview will last no more than an hour.

Before we get started, do you have any questions? Perhaps you could go find your self-test, if you still have it.

**Interview:**

**I’d like to talk with you about this [disease or risk factor] self-test. First of all, I’d like to know about your experiences. What was it like for you doing this self-test?**

🡪 Start with an open question, to get a rough idea of how consumers use the test and of the issues that particularly concern them (e.g. problems performing the test or interpreting the results).

This may raise issues that you can elaborate on later or that you hadn’t thought of.

**OK, you’ve now given me some impression of your experiences. I would now like to discuss why you decided to do this particular test.**

1. REASONS FOR TESTING

- 1. Why did you do this test?
  - 1.1 Had you done it before?
  - 1.2 What expectations did you have before the test? Did you think about what the test result would mean?
  - 1.3 Did you consider other options for testing (such as a different self-test or going to see a doctor)?

**We’ve now talked about your reasons for doing a self-test and your expectations about the test. Your main reason for doing a self-test was….**

**I’d now like to continue with a few questions about the user-friendliness of the test and whether the test was easy to work with and easy to understand.**

2. USER-FRIENDLINESS, INTERPRETATION AND RELIABILITY

- 2.1 Which test did you do (total cholesterol, HDL)?
- 2.2 How did it go?
  - 2.2.1 Was the test easy to use or did you have any problems doing it?
  - 2.2.2 How did you solve these problems? Did you perhaps ask someone to help you do the test?
- 2.3 Was it clear to you what the test result meant for you?
- 2.4 Did you think the test result was reliable?
  - 2.4.1 Why/Why not?
- 2.5 Would you consider doing a self-test again in the future? For cholesterol or for other disorders?
  - 2.5.1 Why / Why not?

**You’ve just told me that you thought this test was easy / difficult to use. We also talked about the test result and what it meant for you. I’d now like to continue with a few questions about what you did after doing the self-test.**

*3. FOLLOW-UP*

- 3.1 What was the result of the test? (It’s no problem if you’d rather not discuss this.) The following questions are about what you did with the test result.
- 3.2 What were your thoughts when you saw the result?
  - If the result was normal: did this reassure you?
  - If the result was abnormal: were you concerned about the result?
- 3.3 What did you do next after seeing the test result?
  - Did you make any changes in your life (for instance try and lead a healthier life)?
  - Did you discuss the test result with a doctor or another type of care provider? Why?
  - How did this consultation go?
- 3.4 Are you currently being treated for a high cholesterol level or for a cardiovascular disease? (It’s no problem if you’d rather not discuss this.)

**We’ve just discussed what you did after getting the test result. Finally, I’d like to ask you a few questions about the information you used when choosing and doing the test.**

4. INFORMATION

- 4.1 What information did you use when you decided to buy this test?
  - What did you think of this information?
- 4.2 What information did you use when you were actually performing the test?
  - What did you think of this information?
- 4.3 What information did you use when you interpreted the test result (in other words to decide what you had to do with the result)?
  - Did the patient information leaflet that came with the test give you any advice about what to do with the result?
  - What did you think of this information?
- 4.4 Was it clear to you whether this test was suitable for you?
  - Was it clear to you what exactly this test measures; could you explain this in your own words?
- 4.5 Did the packaging of the test or the patient information leaflet that came with it tell you anything about the reliability of the test?
  - Do you think this information is important?
- 4.6 What information did you think was lacking?
  - What information do you think it would be important to have before you buy a test, to help you make the right choice?
- 4.7 Where or from whom would you prefer to get this information? For instance, the Internet, your family doctor, a chemist, etc.

*I’d like to thank you very much for this interview. I’d like to emphasize once more that what you told me will be treated in the strictest confidence. If you should have any further questions or comments, don’t hesitate to contact me. Do you perhaps have any questions now? Or are there things we haven’t discussed but which you feel are important to mention?*

*I have one more question to ask you. It’s possible that when I’m analyzing and describing this interview I come across some things that make me wonder whether I really understood you correctly. Or that there are certain experiences on your part that I’d like to know more about. In that case, would you mind if I phoned you? And would you be willing to give us feedback after we have drawn our conclusions? Would you allow us to contact you about that?*
