# Supplementary material for: Improving behaviour in self-testing (IBIS): Study on frequency of use, consequences, information needs and use, and quality of currently available consumer information (protocol)
Source: BMC Public Health. 2010 Aug 3;10:453. doi: 10.1186/1471-2458-10-453 (PMC2919481; doi:10.1186/1471-2458-10-453)
Supplement: Additional file 4 — Interview protocol consumers who intend to use a self-test. Semi-structured topic guide used for the interviews which addresses the reasons to use a self-test, the user-friendliness of the test, the interpretation and perceived reliability of the test result, the information needs and use, and follow-up actions based on the test result. [file 1471-2458-10-453-S4.DOC]

**Interview protocol consumers who intend to use a self-test**

First of all I’d like to thank you very much for your willingness to take part in this interview. Let me first explain to you what this study is about. We work at Maastricht University and are studying the use of self-tests by consumers. The aim of our study is to examine the strong and weak aspects of self-tests. We also want to know why people may hesitate about doing self-tests. The ultimate goal of the study is to develop better information for a number of self-tests. To achieve this, we need help from people like you, which is why we are here today.

One year ago, you received and completed questionnaires about self-tests from Flycatcher, and you indicated then that you might do a self-test for [disease or risk factor] or [disease or risk factor] at some time in the future.

I would like to talk with you about self-tests. We will discuss three aspects, namely your opinion about self-tests, what you think it would be like to do a self-test and what information you would like to have before doing a self-test.

What I want to hear from you is your opinion, so there are no correct or incorrect answers. What I’m interested in is your views. Of course what you say in this interview will be treated confidentially. With your permission I’s like to tape this interview. All your information will be processed anonymously, so nobody could trace it back to you.

The interview will last no longer than an hour.

Before we get started, do you have any questions?

**Inleiding**

**Interview questions**

Could you tell me your opinion about self-tests?

1. You indicated on the questionnaire that you might / would probably / would definitely do a self-test at some time in the future. What’s your opinion about that now?

2. What self-test might you want to do in the future? Any other tests?

2a Why this test / these tests in particular?

3. What are the reasons why you haven’t done this test / these tests so far? / Why did you never actually do so?

4. What do you think are the advantages of doing a self-test / What appeals to you about doing a self-test?

4a. Why? Are there any other advantages?

4b. Do you find it annoying to have to wait for a test result from your family doctor / the hospital? / You sometimes have to wait a long time to get a test result from your family doctor…

4c. Can you tell me how long you think doing a self-test would take?

5. We were just talking about the advantages of self-tests; do you think there are also disadvantages?

5a. Why?

🡪 Can you think of other disadvantages?

5b. How much money would you be prepared to spend on a self-test? What’s that figure based on?

5c. Can you tell me to what extent these cost aspects would affect your choice?

5d. And what if a test was completely free of charge?

5e. Do you think it’s important that a doctor is present when you get a test result?

6. Do you think the results of self-tests are reliable? Why?

Optional: Do you think the test-result could be different if you have the test done at your family doctor’s or at the hospital?

11. How do people around you / How does your partner think about self-tests?

11a. Can you tell me if there are people around you who have ever done a self-test?

11b. To what extent does it matter to you what people around you think about this?

- - Why?

11c. To what extent does it matter to you what your family doctor thinks about self-tests?

**You haven’t done a self-test yet, but I would like to know what you think it would be like to do a self-test:**

12. Why do you think it would be difficult / easy to do a self-test?

- Would you need any help if you had to take a blood sample from your finger?
- Are you afraid it would hurt?
- Are there any other aspects of self-tests that you would worry about?

13. Suppose you do a self-test and the result is abnormal, what would you do?/

Suppose the result of a self-test shows there’s something wrong?

13a. Suppose the test result is favorable, in other words, it says there’s nothing wrong, would that reassure you? What would you do?

**I would now like to continue with some questions about the kind of information you would want to get before, during and after doing a self-test.**

14. Where would you prefer to buy or order self-tests?

15. Suppose you’d want to buy a self-test, what information would you like to have before deciding to buy it?

16. How (or from whom) would you like to get information about self-tests?

17. What do you expect from the information offered in the patient information leaflet that came with the test?

17a. What kind of information would you like to see in a patient information leaflet?

17b. How much information would you like to get about the disorder for which the test is intended?

17c. What do you think the instructions for use should look like?

To what extent are pictures in patient information leaflets important to you?

17d. What information would you want to get once you’ve started the test?

18. How would you go about looking for a self-test?

18a Does this seem easy or difficult to you?

(Do you have access to the Internet?)

18b. Suppose you wanted to look for a self-test on the Internet, how would you go about that?

Do you think this would be easy or difficult?

19. If you have ever visited a website offering self-tests, what did you think about it?

19a. Can you remember which website this was?

20. What do you think a website on self-tests should look like?

20a. What kind of information would you want to find on such a website? Why these particular aspects?

20. Would it make a difference to you who is offering a self-test?

For instance, would you be more likely to do a self-test if it was offered by the national kidney foundation than if it was offered on a website?

If so, why / If not, why not?

21. Have you ever heard of preventive consultations? If so, are there reasons why you would rather do a self-test/preventive consultation than a preventive consultation / self-test?

*I’d like to thank you very much for this interview. I’d like to emphasize once more that what you’ve told me will be treated in the strictest confidence. If you should have any further questions or comments, don’t hesitate to contact me. Do you perhaps have any questions now? Or are there things we haven’t discussed but which you feel are important to mention? Are there any other comments you’d like to make?*

*I have one more question to ask you. It’s possible that when I’m analyzing and describing this interview I come across some things that make me wonder whether I really understood you correctly. Or that there are certain experiences on your part that I’d like to know more about. In that case, would you mind if I phoned you? And would you be willing to give us feedback after we have drawn our conclusions? Would you allow us to contact you about that?*
